# Initial clinical experience of Stereotactic Body Radiation Therapy (SBRT) for liver metastases, primary liver malignancy, and pancreatic cancer with 4D-MRI based online adaptation and real-time MRI monitoring using a 1.5 Tesla MR-Linac

**DOI:** 10.1371/journal.pone.0236570

**Published:** 2020-08-07

**Authors:** William A. Hal, Michael W. Straza, Xinfeng Chen, Nikolai Mickevicius, Beth Erickson, Chris Schultz, Musaddiq Awan, Ergun Ahunbay, X. Allen Li, Eric S. Paulson

**Affiliations:** Department of Radiation Oncology, Medical College of Wisconsin, Milwaukee, WI, United States of America; University of Nebraska Medical Center, UNITED STATES

## Abstract

**Purpose/Objectives:**

Recently a 1.5 Tesla MR Linac has been FDA approved and is commercially available. Clinical series describing treatment methods and outcomes for upper abdominal tumors using a 1.5 Tesla MR Linac are lacking. We present the first clinical series of upper abdominal tumors treated using a 1.5 Tesla MR Linac along with the acquisition of intra-treatment quantitative imaging.

**Materials/Methods:**

10 patients with abdominal tumors were treated at our institution. Each patient enrolled in an IRB approved advanced imaging protocol. Both daily real-time adaptive and non-adaptive methods were used, and selection criteria are described. Adaptive plans were based on pre-beam motion-averaged or mid-position images derived from respiratory-correlated 4D-MRI. Quantitative intravoxel incoherent motion diffusion-weighted imaging and T2 mapping were acquired during plan adaptation. Real-time motion monitoring using cine MRI was performed during beam-on.

**Results:**

Median patient age was 68.2, five patients were female. Tumor types included liver metastatic lesions from melanoma and sarcoma, primary liver hepatocellular carcinoma (HCC), and regional abdominal tumors included pancreatic metastatic lesions from renal cell carcinoma (RCC) along with two cases of recurrent pancreatic cancer. Doses included 30 Gy in 6 fractions, 33 Gy in 5 fractions, 50 Gy in 5 fractions, 45 Gy in 3 fractions, and 60 Gy in 3 fractions, depending on the location and clinical circumstances. Treatments were feasible and were successfully completed in all patients without significant acute toxicity, technical complications, or need for back up CT based treatment plans.

**Conclusions:**

We present a first clinical series of patients treated for pancreatic tumors, primary liver tumors, and secondary liver tumors with a 1.5 Tesla MR Linear accelerator using adapt-to-position and adapt-to-shape strategies. Treatments were well tolerated by all patients. Acquisition of fully quantitative MR imaging was feasible during the course of the treatment delivery workflow without extending overall treatment times.

## Introduction

Magnetic resonance imaging (MRI) guided radiation therapy is rapidly being introduced around the world [1]. The integration of real time MRI guidance offers multiple theoretical advantages over traditional CT-based image guidance [2,3]. For several years, a 0.35 Tesla MRI equipped linear accelerator has been used to treat patients, with multiple published clinical outcomes [4–8]. More recently a 1.5 Tesla magnetic resonance (MR) equipped linear accelerator (MR-Linac) has become a commercially available treatment device for the delivery of external beam radiation therapy [2,9,10]. The use of a diagnostic field strength MRI offers excellent soft tissue contrast. It also presents an opportunity for routine collection of quantitative MR image- based biomarkers of treatment response [11–14]. However, it also presents uncertainties and challenges with regard to treatment feasibility and safety. Higher field strength introduces the possibility of the electron return effect (ERE), for which modeling is attempted but still holds some uncertainties [15]. This is particularly true for patients treated with upper abdominal tumors where dynamic air/tissue interfaces are a common occurrence. Moreover image acquisition is challenging in the upper abdomen given the considerable movement present. In January of 2019 the radiation oncology department at the Froedtert and Medical College of Wisconsin began clinical treatment using a 1.5 Tesla MR-Linac. To our knowledge a clinical outcomes description of upper abdominal tumors, including both pancreatic, primary and secondary hepatic tumors treated with stereotactic body radiation therapy (SBRT) has not been previously published. Our aim was to report the clinical feasibility, along with acute toxicity of treatment using a 1.5 Tesla MR-Linac in the first ten patients treated with SBRT in the upper abdomen. Moreover, we aim to present preliminary institutional criteria as to how patients are selected for different treatment types that are available for treatment on the 1.5 Tesla MR-Linac, including adapt to position (ATP) versus the adapt to shape (ATS) work flows [16]. Finally, we include institutional safety checklists and planning criteria used for treatment.

## Methods and materials

### Patients, protocol and device

Patients were recruited if they underwent treatment on the 1.5 Tesla MR-Linac (*Unity*, *Elekta AB*, *Stockholm Sweden*) at the Froedtert and Medical College of Wisconsin from January 2019-June 2019. The device is FDA approved and considered a standard of care option for treatment with radiation therapy. Each patient consented to enrollment on an institutional based IRB approved imaging study. Patients provided written informed consent for clinical and technical data to be obtained, along with additional quantitative and 4D MR images to be acquired during the course of treatment with radiation therapy. All patients included had either primary liver tumors, secondary liver metastatic tumors, primary pancreatic cancer, or secondary metastatic lesion to the pancreas. Full patient characteristics, tumor types treated, additional research MR imaging sequences acquired, and prescription doses are reported in Table 1. This study was approved by the Medical College of Wisconsin Institutional Review Board (PRO00022903).

**Table 1:** Patient characteristics.

	Tumor Site	Age	Sex	RT Dose	ATP/ATS	Median Total Treatment time (min)	Quantitative images acquired during treatment
Patient 1	Liver met (ocular melanoma primary)	47	F	45 Gy / 3 frac	ATP	49	IVIM, CPMG, B0 Maps
Patient 2	Pancreatic CA Recurrence	77	F	30 Gy / 6 frac	ATS	52	IVIM, CPMG, B0 Maps
Patient 3	HCC	66	M	40 Gy / 5 frac	ATS	62	IVIM, CPMG, B0 Maps
Patient 4	HCC	76	M	45 Gy / 5 frac	ATP	45	IVIM, CPMG, B0 Maps
Patient 5	Liver met (ocular melanoma primary)	93	M	50 Gy / 5 frac	ATP	42	IVIM, CPMG, B0 Maps
Patient 6	Pancreatic CA	72	M	33 Gy / 5 frac (panc)	ATS	67	IVIM, CPMG, B0 Maps
	(primary adenocarcinoma and solitary liver met from pancreas)				ATP	49	
				50 Gy / 5 frac (liver)			
Patient 7	RCC met to pancreas	67	M	25 Gy / 5 frac	ATS	74	IVIM, CPMG, B0 Maps
Patient 8	Liver met (sarcoma), 2 lesions,	60	F	60 Gy / 3 frac (seg. 4A/B)	ATP	56	IVIM, CPMG, B0 Maps
					ATP		
	4A/B and 5/8 lesion			50 Gy / 5 frac (seg. 5/8)			
Patient 9	Liver met (melanoma), 2 lesions, Seg 8 and Seg 3	65	F	60 Gy / 3 frac (seg. 8)	ATP	51	IVIM, CPMG, B0 Maps
					ATS	64	
				50 Gy / 5 frac (seg. 3)			
Patient 10	Pancreatic CA recurrence	59	F	30 Gy / 5 frac	ATS	57	IVIM, CPMG, B0 Maps

RCC- Renal Cell Carcinoma, HCC- Hepatocellular Carcinoma, CA- Cancer

Met- metastases, Frac- Fractions, ATP- Adapt to position, ATS- Adapt to shape, min- minutes, CA- Cancer, HCC- Hepatocellular Carcinoma, RCC- Renal Cell Carcinoma, IVIM- intravoxel incoherent motion, CPMG- Carr-Purcell-Meiboom-Gill.

### Simulation imaging and treatment planning

For each patient, a series of CTs and MRIs were acquired during RT simulation. A reference plan was created based on the simulation CT (reference CT) registered with the simulation MRI set. IV contrast was used at the time of both MR and CT simulation. For patients with liver metastasis, a mid-position CT created from 4DCT was registered to the IV contrast CT, along with a fat-suppressed T2 MR, and an MR using Eovist with 20 and 30 minute delay images. Motion management was accomplished using an internal target volume (ITV) approach, which was created based on the 4D CT simulation. In addition, 4D MRI was used to confirm adequate coverage of moving targets. PTV margins ranged from approximately 0.3 to 0.5 cm. Reference plans for treatment on the MR Linac and back up plans for treatment on a conventional Linac system that use CT based image guidance were created using Monaco treatment planning software (*Elekta AB*, *Stockholm*, *Sweden*).

### Day of treatment procedures and intra-treatment imaging

**Fig 1:**
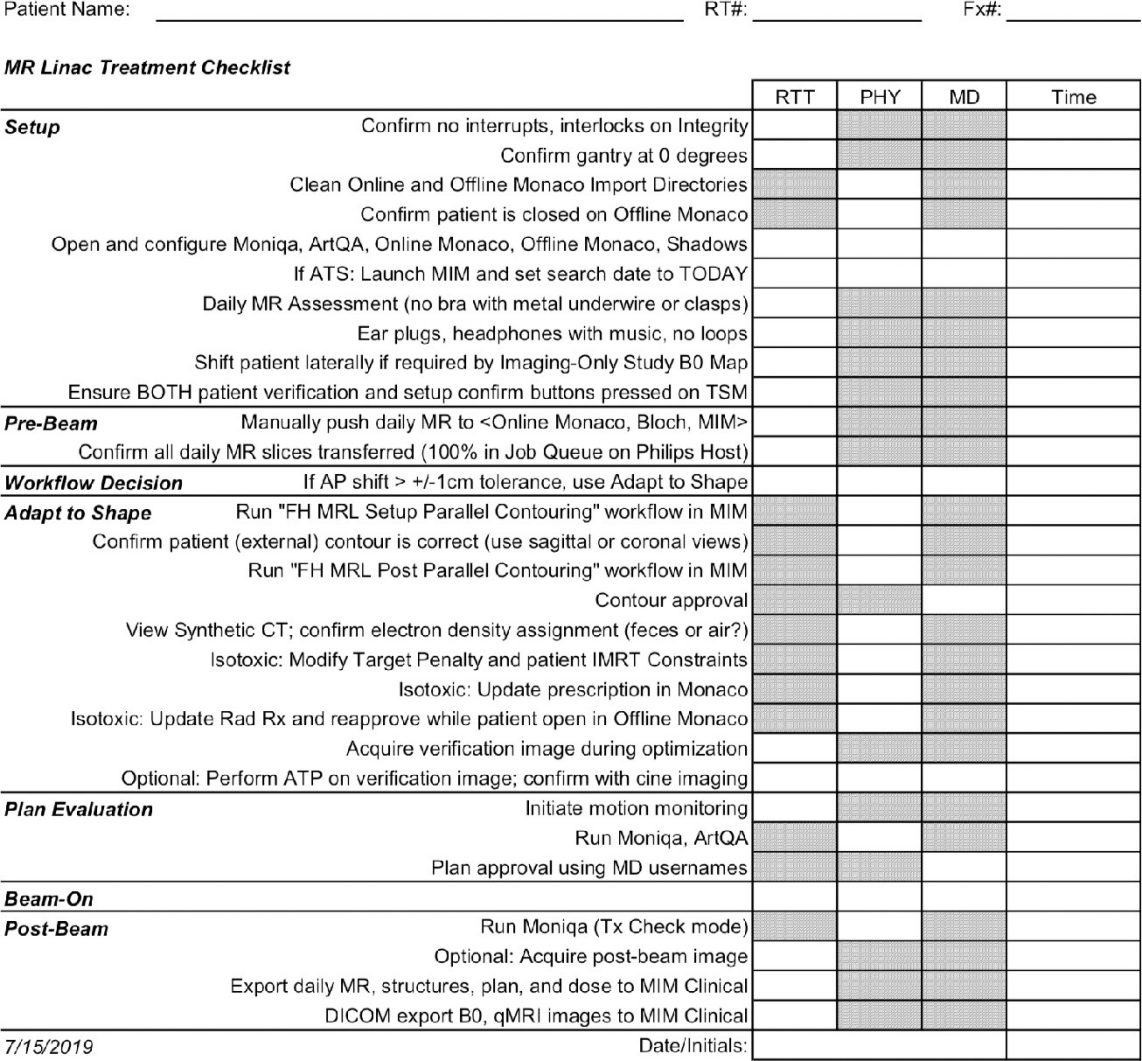
Institutional checklist used for treatment approval and workflow on the MR Linac.

A treatment checklist was designed for use during daily treatment and was used before and during each treatment procedure (Fig 1). This checklist presents a detailed step by step procedure of daily imaging and has been made available at the following public website (mrl.mcw.edu). Pre-beam T1 or mixed T2/T1-weighted 4D MRIs were acquired. Motion- averaged or mid-position images were reconstructed using a separate high-performance reconstruction server positioned within the MR-Linac network. Quantitative intravoxel incoherent motion (IVIM) diffusion-weighted imaging (DWI) and T2 mapping were acquired concurrently during plan adaptation with either adapt-to-position (ATP) or adapt-to-shape (ATS) workflows [16]. ATP is used to correct for translational shifts by adjusting beam apertures and weights, while ATS is performed to account for all interfraction changes by re-optimizing the plan based on the MRI of the day. These treatment strategies have been previously described [16]. Real-time cine MRIs acquired in three perpendicular planes through the PTV center of mass were used to monitor the target during radiation delivery.

## Results

A total of ten patients with upper abdominal tumors have been treated with SBRT at Froedtert and the Medical College of Wisconsin. Patients ranged in age from 47 to 93, with a median age of 66.5 years. A total of four patients had metastatic disease to the liver, two patients had primary hepatocellular carcinoma, three patients had primary pancreatic adenocarcinoma, and one had metastatic renal cell carcinoma (RCC) to the pancreas. The median treatment time, measured as total table time experienced by the patient, was 54 minutes. For patients treated with the ATP treatment strategy, the median total treatment time was 49 minutes and for those patients treated with the ATS the median total treatment time was 64 minutes; these were found to be statistically different (p-value = 0.004), Table 1.

All patients had back up plans created on CT- based linear accelerators. This was done in the event of machine failure or technical issues prohibiting successful treatment completion. Each of the required dosimetric constraints was reviewed and met for each of the backup CT based treatment plans. Plans quality was comparable with few appreciable differences in this cohort. However, all plans were successfully completed on the MR-Linac, and the back-up plans were not used. Additional MR- based research imaging sequences were also successfully acquired within the timeframe needed for treatment. Table 1 summarizes research MRI sequences that were acquired.

**Fig 2:**
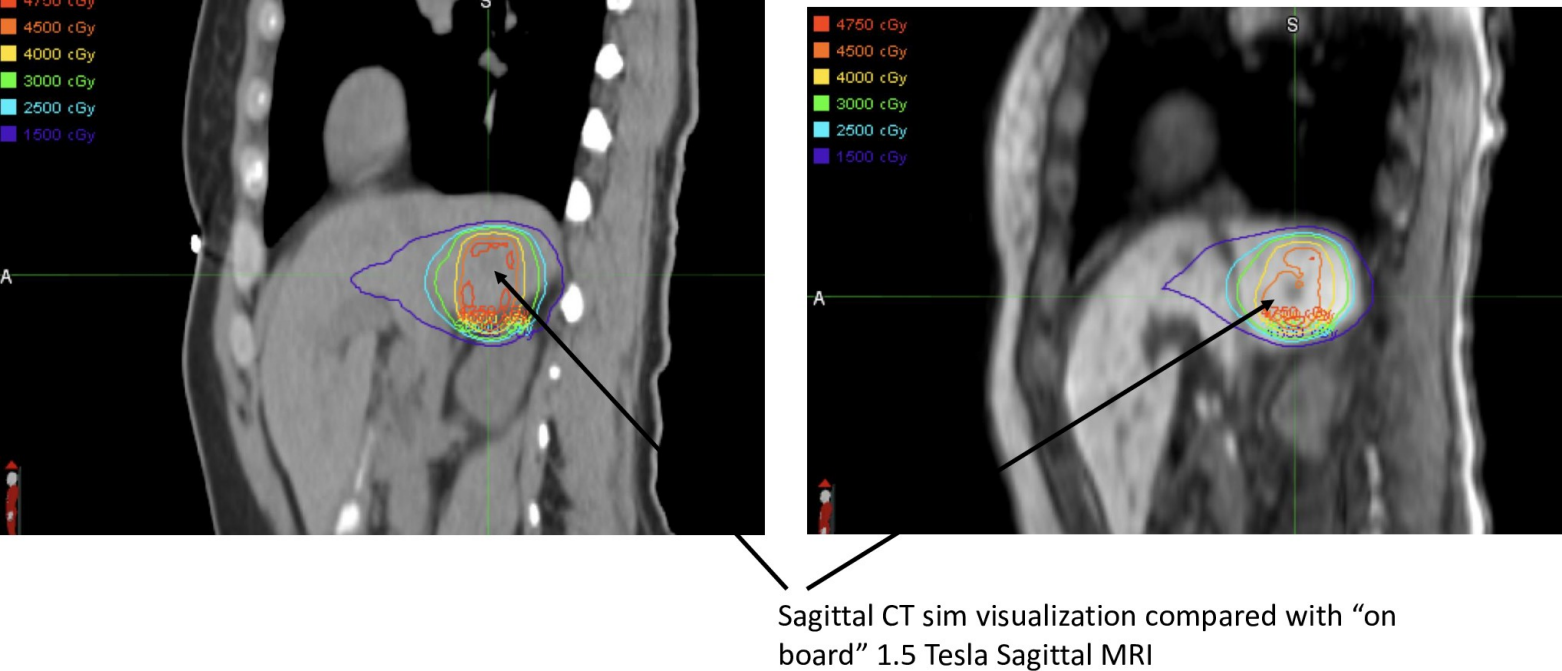
Improved visualization with 1.5 Tesla MRI as compared with CT on rails.

Patients were treated with either ATP or ATS strategies [16]. Institutional selection criteria for treatment on the MR Linac, along with each of these treatment strategies are presented in detail in Table 2. These criteria represent a consensus of treating physicians and involved physics staff. Available imaging was felt to be helpful to identify tumors, particularly in the liver, as compared with CT. An example of such imaging from a patient treated with a secondary liver tumor can be seen in Fig 2.

**Table 2:** Selection considerations for MR-Linac treatment and ATP versus ATS.

MR-Linac as compared with CT based image guidance	CT “on rails” treatment selection	MR Linac treatment selection
	• Lesion is well visualized on non-contrast CT • Lesion is not close (> 1 cm) from a highly mobile and radiosensitive critical normal	• Lesion is difficult/impossible to see using the non-contrast CT • Lesion has minimal movement, and can be safely managed with an ITV approach • Lesion is close to a mobile normal structure that is radiosensitive (luminal GI structures) • Patient is a good clinical trial candidate and interested in trial participation with a tumor that is amenable to MR biomarker based research
MR-Linac Treatment Strategy selection	ATP Treatment selection	ATS Treatment Selection
	• Absence of critical structure with significant mobility in close proximity to treatment volume • Consistent daily positioning • Low chance of daily size variations	• Critical normal structure in close proximity (3–5 mm) to tumor with significant mobility (small bowel, colon, stomach, rectum) • Rapidly changing tumor size • Unexpected daily variation • Considerable variability in daily position of normal organs • Close proximity to air cavity

ATS- “Adapt to shape”, involves the creation of a new contour set daily ATP- “Adapt to position”, plan is re-calculated, but there is not a recontouring of normal organs

**Fig 3:**
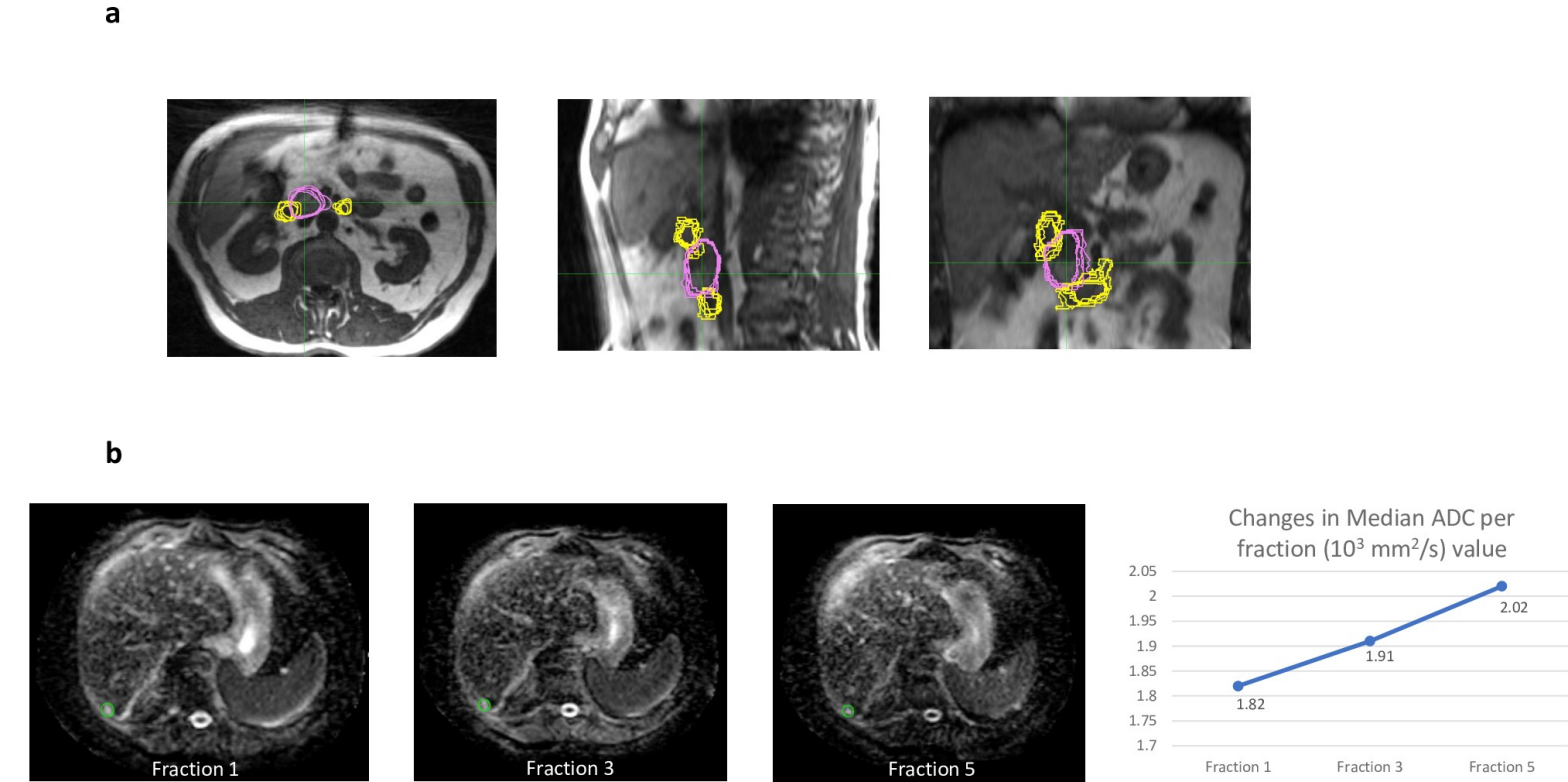
a. Aggregate contours for pancreatic ATS case illustrating daily small bowel and pancreatic CTV position differences, CTV (pink), small bowel (yellow). b. ADC measured during treatment with radiation, with increase in median ADC value during SBRT.

In the ATS workflow, normal structures are contoured by the attending radiation oncologist based on the daily MRI acquired, thus the daily changes of these structures were accounted for with a new plan. Fig 3A visually highlights the changes in contours of the small bowel (yellow), that were seen in close vicinity to the pancreatic primary tumor, contoured on a daily basis before treatment delivery. In addition, Fig 3A shows changes in the daily position of the CTV (pink). Fig 3B presents an example of quantitative MR biomarker intra-treatment images. These were acquired while the ATS process was taking place, with no added table time for the patient. The ability to measure accumulated dose during a course of treatment on the MR Linac, accounting for normal organ movement, is currently absent and therefore detailed dosimetric comparisons (and possible advantages or absence of advantages) between these treatment strategies are not available in this analysis.

All treatments were well tolerated, although at this time the median follow up is short, approximately 7.2 months, so reporting of late toxicities is limited. With regard to acute toxicities, two patients did experience grade 2 skin toxicities during treatment felt to be within expected toxicity for their given tumor locations and isodose distributions. There were no acute grade 3 or higher toxicities experienced during the treatment course. One patient did experience a late grade 3 toxicity, specifically this was a patient with recurrent pancreatic cancer, treated with a repeat course of radiation therapy, who subsequently had an infection in the region of her jejunal anastomoses. This was treated with hospitalization and intra-venous anti-biotics. It was unclear if this was related to treatment with radiation therapy. No other late toxicities, grade 3 or higher, have been observed in this cohort of patients. There have been no local recurrences or progression in the treated lesions at this time, however current follow up (7.2 months) is too premature to draw any conclusions regarding local control. General dose constraints applied to each patient are presented in Table 3.

**Table 3:** Dose constraints used for SBRT for abdominal metastatic lesions or primary hepatocellular carcinoma, DVH Criteria, 5 fraction constraints.

*Organ*	*Constraint Metric*
Liver (Liver minus GTV)	*Hepatocellular Carcinoma (Childs Pugh B7 or better liver function)*:
	Liver Volume Receiving < 15 Gy, Goal = 1100 cc, Min Acceptable = 700 cc, or 30% of liver-GTV volume
	Liver Volume Receiving over 10 Gy, Goal < 70%, Min Acceptable = 72%
	*Metastatic Disease to the Liver*:
	3 fraction: Liver Volume Receiving < 15 Gy, 700 cc or 30% of liver-GTV
	5 fraction: Liver Volume Receiving < 18 Gy, 700 cc or 30% of liver-GTV
Stomach	Max dose less than 34 Gy to 0.03 cc, V20 < 20 cc or V26.5 < 5 cc (if V20 < 20 cc is not feasible). V20 is defined as the volume receiving 20 Gy
Duodenum	No more than 1 cc of may exceed 33 Gy, max may not exceed 34 Gy to 0.03 cc. *Recommended*: V20< 20 cc or V26.5 < 5 cc (if V20 < 20 cc is not feasible). V20 is defined as the volume receiving 20 Gy
Colon	Max dose less than 34 Gy to 0.03 cc *Recommended*: V20 < 20 cc
Small Bowel	Small Bowel, max less than 34 Gy to 0.03 cc *Recommended*: V20 < 20cc or V26.5 < 5 cc (if V20 < 20 cc is not feasible)
Spinal Cord	Spinal Cord, max dose less than 15 Gy
Kidney_R	Right Kidney, V12 < 10%, mean less than 10 Gy
Kidney_L	Left Kidney, V12 <10%, mean less than 10 Gy
One Kidney	V10 < 10%

## Discussion

The integration of MR imaging capabilities into linear accelerators presents a novel technological approach to the treatment of patients with radiation therapy [2,9,13,17,18]. There are both challenges and potential novel advantages to this combination. The potential advantages of routine and real-time MRI acquisition may go beyond improvements in the ability to visualize both tumors and normal tissues. The feasibility of imaging and treatment on these devices, particularly in highly mobile regions of the body, requires careful examination. We present the first ten patients treated in our institution for abdominal tumors using a 1.5 Tesla MR Linac. To our knowledge, this is the first published experience including both liver and pancreatic treatments on a 1.5 Tesla MR-Linac device. Moreover, we believe this is the first published experience to have included both ATP and ATS workflows specifically in these upper abdominal treatment locations. We have presented a rather descriptive series with the goal of illustrating the first ten patients that were selected for treatment, and subsequently treated on the MR Linac. The objective of this is not to compare these treatments to CT- based treatment strategies, but rather to illustrate the decision making process, feasibility, safety checklists, and example images from this novel device and treatment strategy. Future research efforts will need to focus on direct comparative clinical outcomes between these various management strategies.

Based on this initial ten patient experience, treatment on the 1.5 Tesla MR-Linac for abdominal tumors and liver tumors appears to be feasible. The attending radiation oncologists reported the available imaging was felt to be particularly useful to visualize GTV’s, as highlighted in Fig 2. In addition, the use of the MR-Linac in this clinical circumstance (Fig 2) allowed for ablative treatment that would have been otherwise difficult secondary to tumor location and poor visualization on a non-contrast CT. Moreover, interventional fiducial placement would have also been difficult in this location. For some of these reasons, the MR Linac- based treatment strategy was felt to offer an advantage over traditional CT-based treatment. However, this perceived advantage is both subjective and non-quantitative. The clinical advantages of MR guidance (if any) over CT- based image guidance need further exploration, prospective evaluation, and quantitative validation. Such detailed comparative evaluation will be enhanced in the future by currently ongoing prospective registry based studies (NCT04075305).

All ten patients were able to successfully complete their treatment without the need to use the backup conventional CT based treatment plans. Using the ATS work flow, cumulative doses to normal organs for one patient were able to be maintained significantly lower than the upper limits of tolerated dose volume histogram (DVH) constraints, and therefore an additional fraction was able to be added. Treatment times, measured as entire table time, were shorter than initially expected for both the ATS and ATP work flows. Our tabulated treatment times are comparable to other published experiences using 0.35 Tesla MR equipped radiation delivery machines [19]. The ATS median treatment time was found to be significantly longer than that the ATP, by approximately 15 minutes. The longest treatment time for all patients included in this cohort was still found to be less than 80 minutes. Selection criteria for each of the ATS, as compared with the ATP, treatment strategies are described in detail (Table 2).

To help to ensure the safety of our treatments, our department created a checklist for use during the actual MR- guided treatment (Fig 1). This checklist has been made publicly available at the following URL, mrl.mcw.edu. It helped to ensure that all necessary safety procedures took place both before, during, and after the radiation treatment. Such a checklist may be helpful to other centers starting treatment with a 1.5 Tesla MR-Linac.

A very intriguing aspect of 1.5 Tesla MR guided radiation therapy is the ability to acquire quantitative functional MR imaging routinely during a treatment course. Such imaging was acquired in each of the ten patients presented in this series, without any added table time for the patient. This was enabled by the fact the image was acquire during the time the treatment plan was being modified and adapted. The ability to routinely acquire diffusion-weighted imaging and other advanced quantitative imaging series during treatment will likely introduce novel MR image- based biomarkers of treatment response and toxicity that can be conveniently acquired during daily adaptive planning and even during delivery of radiation therapy. How these will correlate with disease response, or potentially enable improved patient selection for dose intensification, or de-intensification strategies remains to be demonstrated. However, this novel frontier of routine “biologically adaptive” radiation therapy is certainly exciting to consider.

A second intriguing aspect of the MR Linac is the ability to acquire 4D MR images during the process of treatment planning and contour adaption using a high performance reconstruction computer. There are several reasons why 4D-MRI was selected for treatment. Currently only free-breathing, non-gated treatments, are supported on the Elekta MR-Linac. Breath hold or respiratory-triggered pre-treatment images could result in a systematic offset of anatomy, as compared to free-breathing non-gated treatment deliveries. A 4D MR is helpful in addition to an ITV based approach as the position, shape (including deformation), rotation, and motion trajectories of abdominal tumors can change significantly from day to day. These changes may impact co-registration of pre-treatment images to reference images in ATP or require daily modification to the original target and ITV from the reference plan in ATS. The supplemental 4D-MRI approach provides volumetric information about the daily position, shape, rotation, and motion trajectories of targets and OARs, with image contrast flexibility to optimize visualization of targets. For patients with small respiratory displacements (i.e. superior/inferior motion < 8mm), a motion averaged volumetric image is reconstructed from the 4D-MRI and used as a daily image for plan adaptation in ATP and ATS (along with the binned 4D-MRI used to construct the ITV). For patients with large respiratory displacements (i.e. S/I > = 8mm) a mid-position volumetric image is reconstructed from the 4D-MRI and used as a daily image for plan adaptation in ATP and ATS (again along with the binned 4D-MRI used to construct the ITV). While manual exception gating, in which a radiation oncologist (or therapist) manually pause the beam based on the position of the target on real-time 2D cine images, is technically possible this was not implemented in these cases. This absence of motion management on the MR Linac required careful patient selection to include patients with relatively limited tumor movement that could be safely managed without a gating treatment strategy. Further development of motion management strategies is actively underway and anticipated.

## Conclusions

We present the first series, to our knowledge, detailing treatment for both pancreatic and liver tumors using a 1.5 Tesla MR-Linac, with 4D MRI acquisition. Treatment on this device was found to be both feasible and well tolerated at this early time point. Safety checklists, example images, quantitative biomarker methods, and total treatment table times are presented. Some of the most important considerations when using the 1.5 Tesla MR-Linac for treatment of abdominal tumors include: 1) highly experienced MR physicist involvement, 2) robust quality assurance, 3) input from diagnostic radiologists, 4) close collaboration amongst multiple radiation oncologists, 5) trial runs before beam on, 6) careful patient selection focused on patients who can tolerate positioning and space restrictions, and 7) robust workflow with distinct roles for each team member. Larger cohorts of patients with prospective quality of life, clinical outcomes, and late toxicity rates are needed (and being collected) [20]. Such larger data sets will enable a more detailed understanding of the potential clinical improvements (if any) associated with MR-guided and MR-adapted treatment. Additional investigation to quantify the precise advantage of MR based image guidance as compared to CT based image guidance is critically needed.
