# Correction: Initial clinical experience of Stereotactic Body Radiation Therapy (SBRT) for liver metastases, primary liver malignancy, and pancreatic cancer with 4D-MRI based online adaptation and real-time MRI monitoring using a 1.5 Tesla MR-Linac

**DOI:** 10.1371/journal.pone.0242146

**Published:** 2020-11-04

**Authors:** The PLOS ONE staff

The first author's name is spelled incorrectly. The publisher apologizes for the error. The correct citation is: Hall WA, Straza MW, Chen X, Mickevicius N, Erickson B, Schultz C, et al. (2020) Initial clinical experience of Stereotactic Body Radiation Therapy (SBRT) for liver metastases, primary liver malignancy, and pancreatic cancer with 4D-MRI based online adaptation and real-time MRI monitoring using a 1.5 Tesla MR-Linac. PLoS ONE 15(8): e0236570. https://doi.org/10.1371/journal.pone.0236570
